# Mechanochemical Deep Impact: Delivering Sustainable Synthesis and Hydrogen Innovation

**DOI:** 10.1002/cssc.202502650

**Published:** 2026-02-16

**Authors:** Ken‐ichi Saitow

**Affiliations:** ^1^ Department of Materials Science Natural Science Center for Basic Research and Development (N‐BARD) Hiroshima University Hiroshima Japan; ^2^ Department of Chemistry Graduate School of Advanced Science and Engineering Hiroshima University Hiroshima Japan

**Keywords:** alkoxysilane, ball mill, hydrogen, mechanocatalyst, titanium dioxide

## Abstract

Mechanochemistry in planetary ball mills is a transformative and sustainable chemical process by which mechanical impact is converted into reaction‐driving energy. High‐energy collisions between balls, analogous to meteorite impacts on Earth, generate transient extreme pressures (∼10 GPa) and temperatures (∼1500°C) and supercritical water in microscale “hot spots,” allowing reactions once restricted to high‐temperature or solvent‐intensive laboratory or industrial conditions to proceed. This platform achieves hydrogen evolution efficiencies comparable or superior to electrolysis and even realizes a new phenomenon—room‐temperature thermochemical water‐splitting cycles—without CO_2_ emissions, oxygen separation systems, or external heaters. Furthermore, the mechanochemical activation of TiO_2_ yields photocatalysts with markedly enhanced absorption from the UV to the near‐infrared through defect and polymorph engineering. Beyond energy applications, the direct halogen‐free, HF‐free synthesis of alkoxysilanes provides a green, scalable route to value‐added chemicals with the coproduction of hydrogen at room temperature. These processes exploit abundant or waste materials, operate in compact setups, and consume very little energy, suggesting their potential for distributed fuel generation and sustainable materials manufacturing. Planetary ball milling can therefore offer a generalizable framework for green chemistry, bridging solid‐state reaction engineering with energy conversion and functional materials synthesis to provide practical routes toward low‐carbon, scalable technologies.

## Graphical abstract

Planetary ball milling unlocks sustainable routes to advanced materials and hydrogen. Transient extreme conditions created during collisions drive highly efficient transformations, enabling the synthesis of visible‐light‐active TiO_2_, alkoxysilanes, and hydrogen through impact‐induced mechanochemistry.

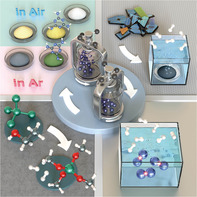

## 1 Introduction

Since ancient times, humans have generated fire by rubbing wooden sticks together or striking flints, unconsciously triggering redox reactions through friction and impact. This primitive technology—in which mechanical energy is converted into chemical reaction energy—represents the earliest form of mechanochemistry. From this origin, mechanochemistry has evolved into a modern scientific discipline concerned with chemical transformations driven by mechanical forces such as compression, shear, and friction. Mechanical operations including grinding, sliding, or deformation can thus initiate distinct reaction pathways, often without heat or solvents, linking human technological history to contemporary sustainable chemistry [1, 2].

An important historical milestone was passed in the 19th century, when Michael Faraday demonstrated that cogrinding metals, such as Zn, with AgCl using a mortar and pestle could trigger reduction and the formation of Ag. This discovery established the conceptual foundation of mechanochemistry, bridging mechanical work and chemical reactivity. Readers seeking a more comprehensive account of the great journey of mechanochemistry—from its ancient roots and medieval alchemical practices in both the East and West to its present resurgence—are referred to several excellent reviews [1, 2, 3, 4, 5, 6, 7, 8, 9, 10, 11].

The 20th century brought a major transformation with the advent of automated mills, which replicate the grinding motion of a mortar and pestle with greater reproducibility and controllability. Recognizing the more recent growing significance of mechanochemistry as a discipline spanning sustainable and energy efficient syntheses for a range of chemicals, the International Union of Pure and Applied Chemistry (IUPAC) identified it in 2019 as one of ten emerging areas expected to shape the future of chemistry [8]. Indeed, major advances have been realized across diverse areas, including organic synthesis [5, 6, 7, 8, 9, 10, 11], transition metal catalysis [1, 7, 8, 9, 10], metal–organic frameworks [5, 12], coordination compounds [6, 8], supramolecular assembly [12], plastic depolymerization [13, 14], pharmaceuticals [15, 16], ammonia synthesis [17, 18], and hydrogen production [19, 20, 21, 22]. More recently, mechanochemistry has been explicitly recognized as tool for achieving many of the United Nations’ 17 Sustainable Development Goals [23, 24].

With the rise of automated mechanochemical techniques, ball mills have become the workhorses of modern mechanochemistry. Two main types of ball mills are commonly employed: shaker (or vibratory) and planetary mills. In a shaker mill, the jar oscillates at a controlled vibration frequency, primarily imparting impact forces to the reagents. This equipment is widely used for homogenizing pharmaceutical solids, organic synthesis, and other mechanochemical reactions, and is closely related to industrial‐scale horizontal and vibratory milling technologies. In a planetary mill, the vessel rotates around a central axis while spinning around its own axis. Such “planetary” motion generates centrifugal forces that either increase the effective gravity (e.g., to ×100 g) or emulate the gravitational conditions of industrial‐scale roller mills, offering a direct connection to scale‐up [5, 7]. Various mill designs, including the abovementioned types, are currently the subjects of active scale‐up efforts in mechanochemistry [25].

Among these instruments, planetary ball mills have attracted particular attention as powerful platforms for high‐energy mechanochemical synthesis [4, 26] such as mechanochemical alloying. Notably, planetary ball milling generates transient, highly localized extreme conditions—pressures up to ∼10 GPa and temperatures approaching ∼1000°C—that can trigger solid‐state phase transitions [1, 2]. Such environments produce high‐pressure polymorphs of Al_2_O_3_ [27, 28], TiO_2_ [29, 30], and intermetallic compounds such as Cu_3_Si [22], whose formation is comparable to the formation of the same compounds in meteorite impact craters [31]. In this context, ball mills have also been employed as laboratory‐scale analogs to explore meteorite‐impact‐like environments relevant to research on the origin of life [32, 33, 34]. Importantly, while many such studies have been conducted under dry conditions, similar high‐energy environments can also arise in the presence of water, enabling the realization of thermochemical water‐splitting cycles and sustained hydrogen production without external heating. Intriguingly, the discovery of this room‐temperature thermochemical hydrogen production process occurred owing to an unexpected incident in which a milling vessel lid was ejected due to rapid gas evolution [20].

This review highlights the emerging role of planetary ball milling as a versatile platform for sustainable chemistry, bridging solid‐state reaction engineering with green energy, sustainable fuels, and sustainable materials. We also discuss the potential implications of mechanochemistry more generally for chemical synthesis applications.

## 2 Activation of Photocatalysts: UV–NIR TiO_2_

Mechanochemistry provides a unique platform to drive solid‐state reactions, phase transitions, and defect formation without the use of solvents or external heating. Beyond conventional materials synthesis, it can unlock reactivities and structures inaccessible through thermal or wet‐chemical routes. To illustrate this emerging paradigm, we focus on the most widely studied photocatalyst, titanium dioxide (TiO_2_), and show how mechanochemical modification activates red‐light‐driven photocatalysis through defect and phase control.

TiO_2_ has long served as a photocatalyst for self‐cleaning, antibacterial, and sterilizing coatings on everyday materials, construction materials, and medical surfaces. However, its activity is typically restricted to UV wavelengths (the bandgap *E*_g_ of anatase is 3.2 eV), which constitutes only 2%–3% of the solar spectrum. Achieving visible‐light‐driven TiO_2_ photocatalysis therefore remains a central challenge for sustainable environmental applications [37].

**FIGURE 1:**
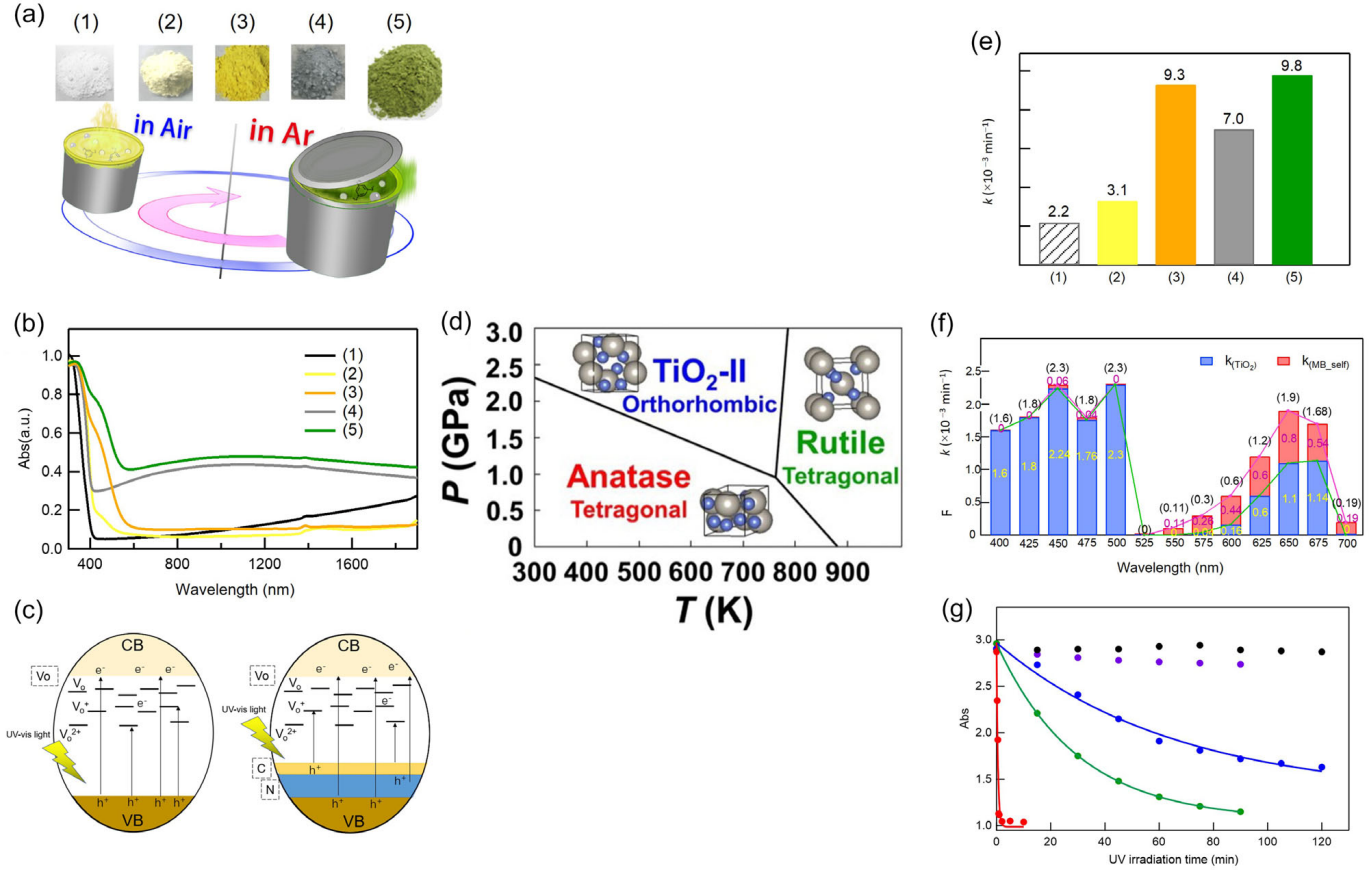
TiO_2_ photocatalyst activation by planetary ball milling. (a) Schematic diagram of sample preparation. Photographs of prepared samples of (1) TiO_2_ (P25) without milling, (2) TiO_2_ milled in air, (3) TiO_2_ milled with melamine in air, (4) TiO_2_ milled without melamine in Ar, and (5) TiO_2_ milled with melamine in Ar. (b) UV–vis–NIR absorption spectra of samples (1)–(5). (c) Schematic diagrams of electronic transitions in samples 4 (left) and 5 (right). Sample 4 (gray TiO_2_) has a large number of midgap states due to oxygen vacancies (V_o_, V_o_^+^, and V_o_^2+^) and sample 5 (green TiO_2_) has states arising from the same vacancies as well as N and C states above the valance band (VB). (d) Phase diagram of TiO_2_. (e) Rate constants *k* illustrating the photocatalytic activities of the different samples under Xe irradiation (*λ* = 350–700 nm). (f) Action spectrum constructed from the *k* values of green TiO_2_ (blue) and dye self‐bleaching (red). (g) Absorbances of dye solutions at 662 nm as a function of UV irradiation time: black symbols—control measurement without irradiation; purple symbols—anatase TiO_2_ without UV irradiation; blue—anatase TiO_2_ before milling; green—as‐purchased P25; red—anatase TiO_2_ after milling. The data shown in panels (a)–(c), (e), and (f) are reproduced with permission from ref. [30] (copyright 2020, American Chemical Society), those in panel (d) are reproduced with permission from ref. [35] (copyright 2019, Taylor and Francis), and those in panel (g) are reproduced with permission from ref. [36] (copyright 2013, American Institute of Physics).

With the aim of overcoming these challenges, mechanochemical ball milling has been employed to prepare disordered TiO_2_ with enhanced photocatalytic performance, for example, for hydrogen evolution [38] and CO_2_ reduction [39]. Among these studies, we developed a simple mechanochemical synthesis route for four different colored TiO_2_ photocatalysts (green, gray, orange, and yellow) by ball milling TiO_2_ with or without melamine for 2 h at room temperature (Figure 1a–c). Tuning via chemical doping was achieved, depending on the milling atmosphere (air or Ar), the balance between oxygen vacancies and Ti^3+^ species, and the concentrations of the N and C supplied from the melamine, as listed Table 1. In parallel, physical structural modification (i.e., physical doping) occurred: the transient gigapascal pressures generated during milling triggered the formation of high‐pressure polymorphs (e.g., TiO_2_ II, as shown in Figure 1d) [30] with narrower bandgaps (*E*_g_ = 1.1–2.7 eV) [35, 40, 41]. The combined effect of these changes resulted in the introduction of midgap states and a reduced bandgap, leading to distinct colors and broad absorption across wavelengths from the UV to the NIR (Figure 1b,c). Characterization revealed that the green and orange TiO_2_, rich in oxygen vacancies and multiphase heterojunctions, exhibited fivefold higher activity than a commercial TiO_2_ sample (P25) (Figure 1e), with red‐light absorption contributing significantly (Figure 1f) [30]. Indeed, P25, consisting of a mixture of anatase (∼80%) and rutile (∼20%) nanoparticles, is widely regarded as a global standard TiO_2_ photocatalyst and is commonly used as a high‐activity benchmark reference. Moreover, even with a UV light source, the photocatalytic activity of mechanically milled TiO_2_ was 130 times higher than that of TiO_2_ anatase prior to milling and 60 times higher than that of a commercially available P25 photocatalyst (Figure 1g) [36].

**TABLE 1:** Synthesis conditions, structures, and properties of colored TiO_2_ photocatalysts.

Sample	Color	Dopant	Milling atmosphere	Phase constituents, %				Direct *E*_g_, eV	Indirect *E*_g_, eV	EF^a^	Number of spins^b^ (10^15^)	Defect density^c^ (10^18^ mol^−1^)
				Anatase	Rutile	TiO_2_‐II	Amorphous					
(1)	White	—	—	76	21	0	3	3.3	3.0	1.0	0	0
(2)	Yellow	—	Air	7	50	18	25	3.1	3.0	1.4	0.0064	0.0256
(3)	Orange	N, C	Air	11	32	28	29	3.2	2.2	4.2	1.036	4.144
(4)	Gray	—	Ar	7	59	11	23	3.1	3.0	3.2	1.24	4.96
(5)	Green	N, C	Ar	10	53	27	10	3.2	2.3	4.5	8.16	32.64

a The enhancement factor (EF) values are a metric to compare the photocatalytic activities before and after milling and are defined in terms of the ratio of the rate constants *k*_after_/*k*_before_, which are shown in Figure 1e.

b The densities were obtained from the number of spins and the weight of the samples. These values were obtained from ESR measurements.

c Defect density as the sum of the V_o_ and Ti^3+^ densities in 1 mol of TiO_2_. Reproduced with permission from ref. [30] (copyright 2020, American Chemical Society).

These findings demonstrate that simple milling can produce colored TiO_2_ photocatalysts with exceptional and stable visible‐light activity, without promoters or metal loadings. The quantitative relationship among disorder, polymorphs, and activity clarifies the origin of visible‐light response and guides rational design of defect‐engineered oxides. Such photocatalysts are resistant to air and moisture [30], and their visible‐to‐NIR responsiveness is expected to enable continuous environmental purification and disinfection even under indoor illumination—an ability recently exemplified by TiO_2_‐based inactivation of SARS‐CoV‐2 [42]. Furthermore, the milled TiO_2_ powders act as extremely effective light‐harvesting antennas (plasmon‐free field enhancement occurs owing to Mie resonance [43]), with EFs of 500 [44] and 2000 [45] having been recorded. Mechanochemical engineering thus offers a scalable pathway to transform a conventional UV photocatalyst into a versatile, visible‐light‐responsive material for sustainable environmental and biomedical applications.

## 3 Activation of Silicon: Turning Waste into Hydrogen

Following the visible‐light activation of TiO_2_, mechanochemical strategies have also opened new pathways for hydrogen production from elemental solids. Among these, Si—an earth‐abundant, nontoxic, and industrially ubiquitous material—has emerged as a candidate for sustainable water‐splitting reactions driven by mechanical energy input rather than heat or electricity.

Si is the second‐most abundant element in Earth's crust and is environmentally benign. The hydrogen yield from 1 g of Si reacting with water theoretically exceeds that from metals such as Al or Mg [21]. Furthermore, waste materials from Si wafer sawing and even discarded Si solar cells can be upcycled for hydrogen production [46], with coproducts including SiO_2_ and Si(OH)_4_ usable as precursors in industrial cement or ceramic production. However, conventional Si‐based hydrogen generation typically requires multiple, time‐consuming pretreatment steps and the use of hazardous chemicals (e.g., HF, SiCl_4_, SiH_4_, or strong alkalis) to create large surface areas and increase reactivity. These processes demand complex handling and protective infrastructure [21].

**FIGURE 2:**
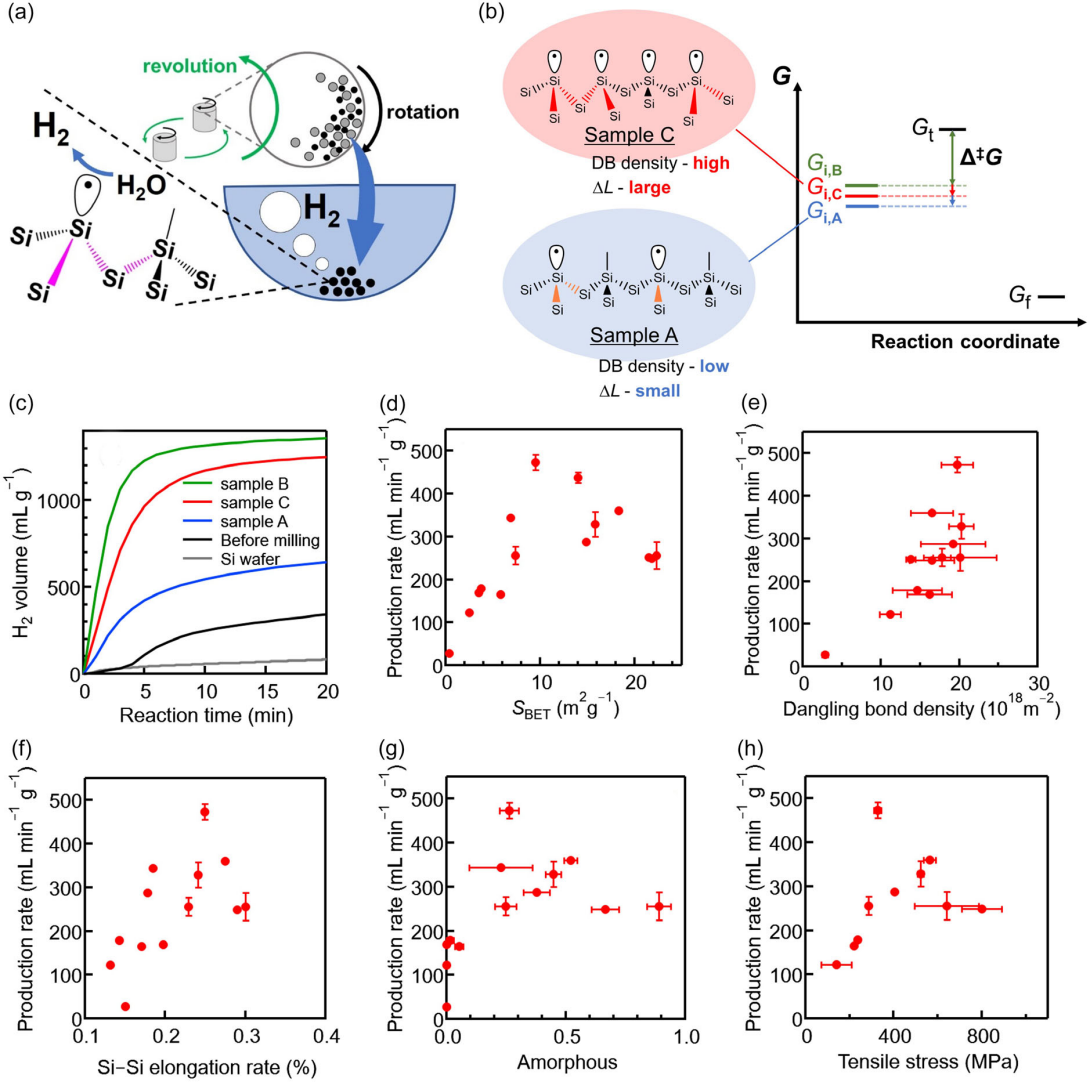
Hydrogen evolution from water using mechanochemically activated silicon particles. Schematics of (a) sample preparation and the reaction between mechanochemically tailored Si particles and alkaline water and (b) the mechanisms of these reactions. Samples A, B, and C were prepared using different milling conditions: 300 rpm for 3 min, 600 rpm for 3 min, and 700 rpm for 20 min, respectively. (c) Time profiles of H_2_ production. H_2_ production rate vs. (d) surface area of Si particles, (e) number of dangling bonds per unit surface area, (f) Si—Si bond elongation rate, (g) amorphous content of Si particles, and (h) tensile stress of Si particles. Reproduced with permission from ref. [21] (copyright 2023, American Chemical Society).

In one study, high‐enthalpy, high‐entropy Si particles were tailored for efficient hydrogen production via ball milling (Figure 2a,b). Well‐defined Si particles—whose surface and internal structures were characterized using eight structural parameters—were reacted with alkaline water at low temperatures (30°C–70°C). The most active particles, obtained after only 3 min of milling at 600 rpm without chemicals, exhibited a high hydrogen evolution rate of 501 mL  min^−1^ g^−1^. These Si particles featured 1% dangling bonds, 0.25% Si—Si bond elongation, 26% amorphous content, and an internal stress of 330 MPa. Thus, this study produced clear structure–property relationships that can be used to guide future design strategies (Figure 2c–h) [21].

**FIGURE 3:**
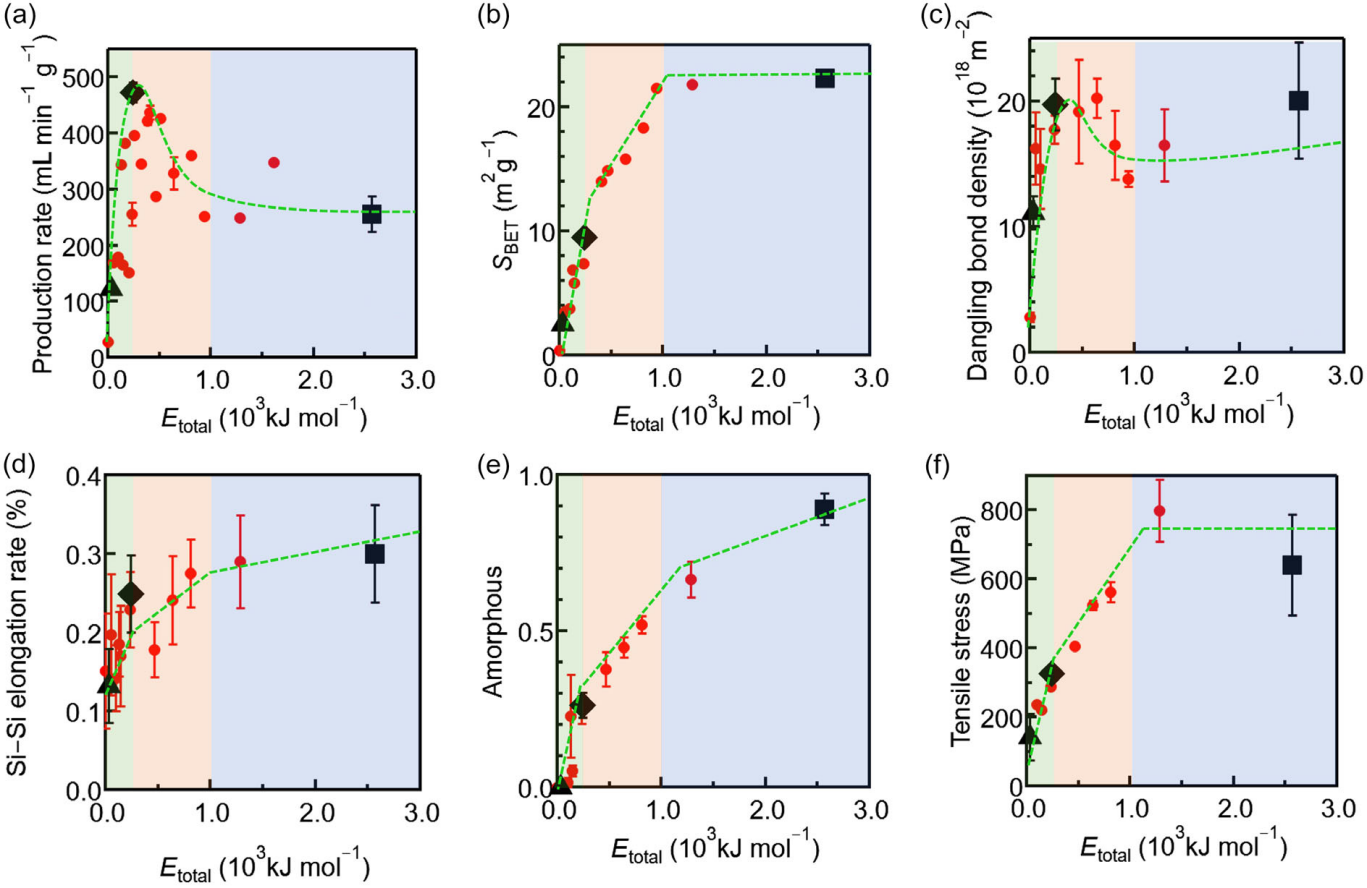
(a) H_2_ production rate, (b) Brunauer–Emmett–Teller (BET) surface area, (c) dangling bonds per unit surface area, (d) Si—Si bond elongation rate, (e) amorphous content, and (f) tensile stress vs. mechanical energy imparted to Si particles during preparation in a planetary ball mill. Black triangles, diamonds, and squares in the three shaded regions correspond to the Si particle samples A, B, and C, respectively, which are described in the caption of Figure 2. Reproduced with permission from ref. [21] (copyright 2023, American Chemical Society).

Importantly, the system achieved an energy efficiency of 2.39 kWh Nm^−3^, superior to that of conventional alkaline electrolysis (5 kWh Nm^−3^), using a compact setup (∼50 cm). Thus, a green, efficient, and cost‐effective route to small‐scale distributed hydrogen generation was demonstrated. Mechanistic investigations further challenged the following two prevailing assumptions: (i) larger surface area always enhances reactivity and (ii) higher mechanical energy invariably improves performance. Instead, the results revealed that the balance between enthalpic and entropic contributions—controlled via the density of dangling bonds per unit surface area—was the key to optimizing hydrogen production efficiency, based on analyses of the structures of the Si particles (Figure 2) and the mechanical energy imparted to them (Figure 3) [21].

## 4 Mechanocatalytic Ti–H_2_O Reaction: Room‐Temperature Thermochemical Water Splitting

Hydrogen is a carbon‐free energy carrier central to the development of sustainable societies, serving as a clean fuel for power generation and a feedstock for ammonia, refining, chemicals, and steel. However, about 95% of commercially available hydrogen is still derived from fossil fuels via steam reforming of natural gas or coal, which requires high temperatures (650°C–1000°C) and produces substantial amounts of CO_2_. Therefore, CO_2_‐free, energy‐efficient hydrogen production methods are urgently needed. Current efforts focus on electrolysis and photocatalysis, but both of these still require energy‐intensive infrastructure, posing challenges for cost reduction, footprint minimization, and widespread deployment [47, 48, 49].

**FIGURE 4:**
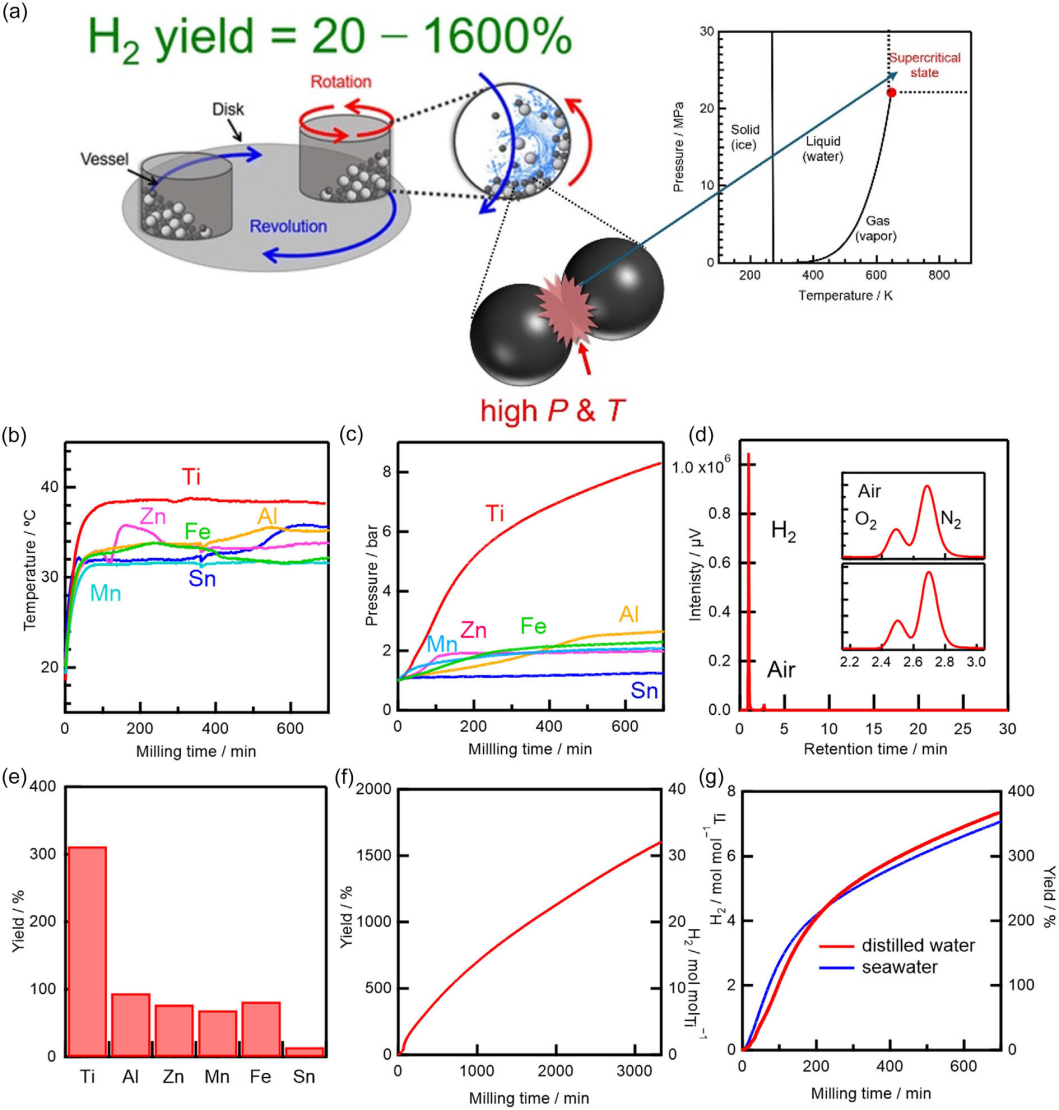
(a) Schematic of thermochemical water‐splitting cycle proceeding in transient supercritical water between colliding balls. (b–g) Experimental observation of mechanochemical metal–water reactions triggered by planetary ball milling: (b) temperature and (c) pressure of the gas in the vessel measured in situ during milling. (d) Gas chromatogram of the gas generated. (e) H_2_ yields after 600 min of milling various metals. (f) Yield (left axis) and amount (right axis) of H_2_ produced by the mechanochemical Ti–water reaction. (g) H_2_ produced during mechanochemical reactions between Ti and distilled water or seawater. Reproduced with permission from ref. [20] (copyright 2024, Royal Society of Chemistry).

Our group accidentally discovered an alternative, purely mechanochemical route to hydrogen production using a planetary ball mill, during nanoparticle synthesis experiments conducted in water. In some trials, the generated H_2_ pressure became so high that the vessel seal ruptured, and the cover was blown to the laboratory ceiling. This unexpected event redirected our research focus toward hydrogen production under controlled conditions, and we were subsequently able to demonstrate room‐temperature thermochemical water‐splitting cycles (Figure 4a) [20].

When water and metal powders (Al, Ti, Zn, Fe, and Mn) were milled together, hydrogen was efficiently generated, without CO_2_ emission or oxygen formation, in yields of 70%–100% at 23°C–38°C (Figure 4b–e) [20]. Under standard‐state conditions (25°C, 1 atm), such metal–water reactions proceed at negligible rates because passivation layers hinder the reaction with water [50]. In contrast, milling in water simultaneously breaks the passivation layer and increases the reactive surface area, while collisions between milling balls create localized regions of high temperature and pressure [20]. These microscale impact sites drive reactions that would otherwise be thermodynamically inaccessible.

**FIGURE 5:**
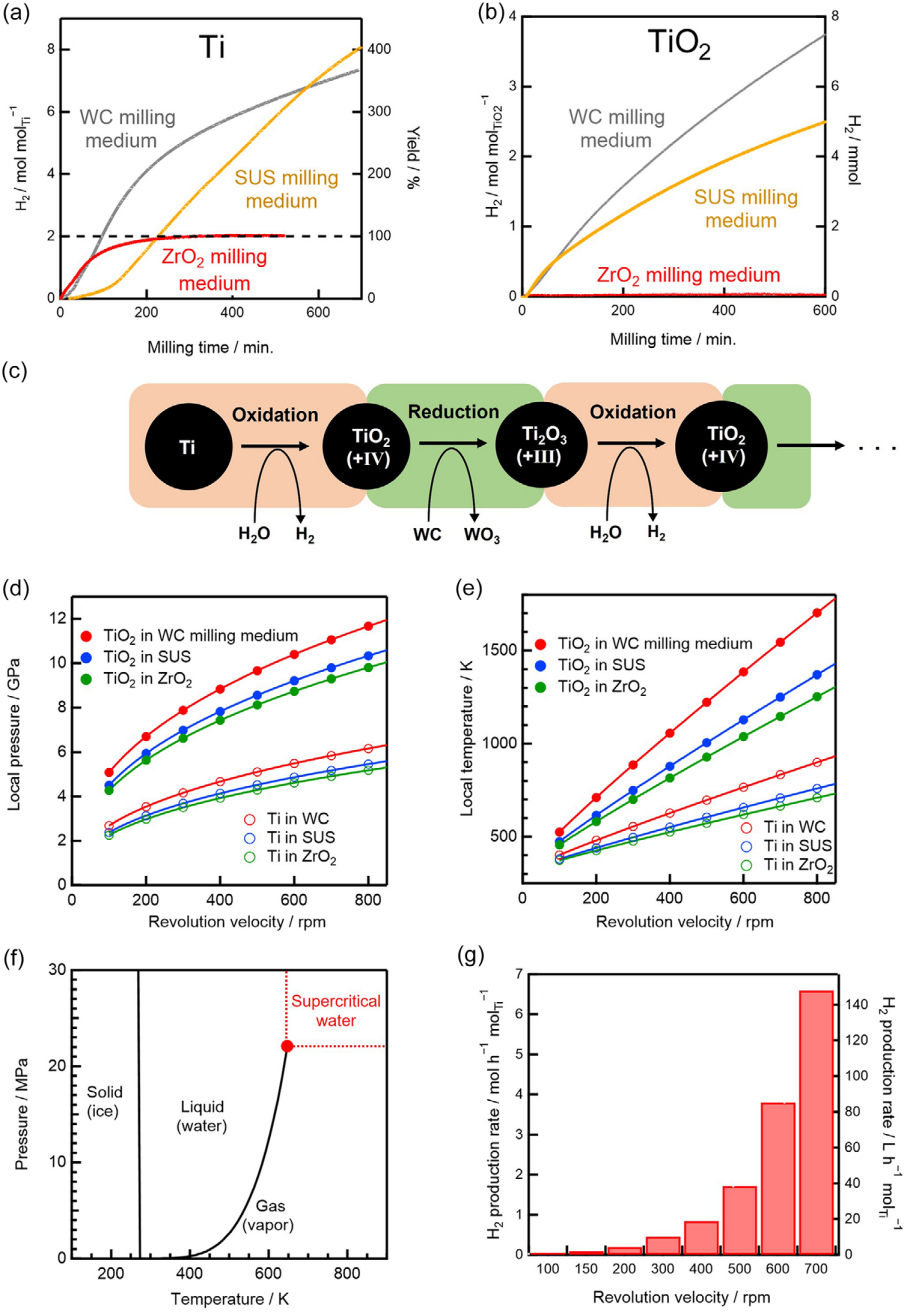
H_2_ production using different milling media during the mechanochemical reactions of (a) Ti and water and (b) TiO_2_ and water. The H_2_ amounts and yields are shown on the left and right axes, respectively. (c) Schematic of thermochemical water splitting cycle for H_2_ production via mechanocatalytic Ti/TiO_*x*_–water reactions, highlighting the role of repeated‐impact‐driven redox cycling. Calculated (d) local impulsive pressure and (e) local impulsive temperature between colliding balls. (f) *p*–*T* phase diagram of water. The red solid circle highlights the vapor–liquid critical point; beyond this point, supercritical water exists. (g) H_2_‐production rate vs. milling revolution velocity. The left and right axes show the H_2_‐production rates in molar (mol h^−1^ mol_Ti_^−1^) and standard‐state volume (L h^−1^ mol_Ti_^−1^) units, respectively. Reproduced with permission from ref. [20] (copyright 2024, Royal Society of Chemistry).

Strikingly, Ti exhibited an apparent hydrogen yield of 1600%, 16 times the theoretical limit for direct Ti oxidation (Figure 4e,f). This extraordinary yield originates from a mechanocatalytic thermochemical water‐splitting cycle, in which Ti first reacts with water to form TiO_2_, and the resulting oxide is reduced back to TiO_*x*_ by the milling medium—tungsten carbide or stainless steel—that acts as a mechanococatalyst (Figure 5a–c). In this sense, the active metal (Ti) functions as a mechanocatalyst, repeatedly undergoing oxidation–reduction cycles, while the milling medium supplies the reducing equivalents through high‐energy mechanical collisions. The cycle continuously regenerates Ti, sustaining hydrogen evolution at room temperature until the available water is depleted [20].

By contrast, it has been reported that hydrogen generation can occur driven solely by friction between milling balls and a vessel in the absence of a metal reactant [19]. However, the amount of H_2_ produced in the reported study was approximately 35 times lower than that obtained with Ti under identical milling conditions [20]. This comparison clearly demonstrates that the presence of Ti dramatically enhances hydrogen production through mechanocatalytic cycling.

Mechanistic analysis revealed that collisions among the 3000 milling balls in the planetary ball mill vessel generate microscale “hot spots” with transient temperatures of 300°C–1500°C and transient pressures of 4–11 GPa (Figure 5d,e). The hotspots are generated at a rate of 2000 per second. These conditions correspond to the water being in a supercritical state (Figures 4a, 5d–g), which accelerates hydrogen production up to 300‐fold [20]. Supercritical water is known to act as a highly reactive oxidant, efficiently releasing H_2_ via water reduction [51, 52]. These localized environments resemble those in deep‐Earth settings, where natural hydrogen forms owing to high‐*p*–*T* water–rock interactions involving supercritical water [53, 54]. It is interesting to note that planetary‐scale geochemical processes and localized events in a “planetary” ball mill seem to share a mechanochemical pathway for hydrogen evolution.

Remarkably, although the bulk temperature remains near 30°C, these transient localized supercritical states [20] allow Ti‐based reactions to proceed that are otherwise only accessible in conventional high‐temperature thermochemical water‐splitting cycles operating at ∼2,000°C water temperatures, such as Ti redox cycles driven by heliostat fields [55]. These high temperatures are typically achieved using large solar concentrators (i.e., heliostat fields located in deserts with footprints of ∼155,000 m^2^) or nuclear reactors [49, 55, 56, 57]. Indeed, comprehensive analyses—including in situ *p*–*T* monitoring, X‐ray diffraction (XRD), X‐ray photoelectron spectroscopy (XPS), and thermodynamic–kinetic modeling—confirmed that hydrogen formation proceeds in microscale regions via a thermochemical mechanocatalytic water‐splitting cycle driven by mechanical impact, with reaction products formed at local temperatures exceeding 400°C. Importantly, even seawater can serve as a feedstock to achieve thermochemical water‐splitting cycle (Figure 4g), yielding >99% pure hydrogen without Cl_2_ or CO_2_ emissions or the requirement for gas purification [20].

Unlike previous mechanochemical studies, where milling merely activated metals before subsequent thermal reactions, this approach enables continuous hydrogen evolution at near‐room temperature through self‐sustaining redox cycling. By harnessing microscale supercritical environments generated by collisions, a new class of localized, transient thermochemical hydrogen production within mechanical reactors is achieved [20].

## 5 Halogen‐Free Alkoxysilane and H_2_ Synthesis at Room Temperature

The versatility of mechanochemical reactions means that their scope extends far beyond hydrogen production. Alkoxysilanes are key precursors for silicones used in medical devices, electronics, coatings, ceramics, and lubricants [58], as well as mesoporous silica [59, 60, 61] and flexible aerogels [62]. Conventionally, they are synthesized by reacting chlorosilanes with alcohols at high temperatures (>250°C), generating corrosive HCl byproducts that complicate purification and damage equipment. Alternatively, direct synthesis using silicon powder and alcohols as starting materials can produce alkoxysilanes with hydrogen as a coproduct, but it also requires high temperatures (>250°C), as well as HF treatment to remove the SiO_2_ passivation layer. Thus, developing a halogen‐free and HF‐free route utilizing mild conditions represents a long‐standing challenge in the search for genuinely green silicon chemistry [22].

**FIGURE 6:**
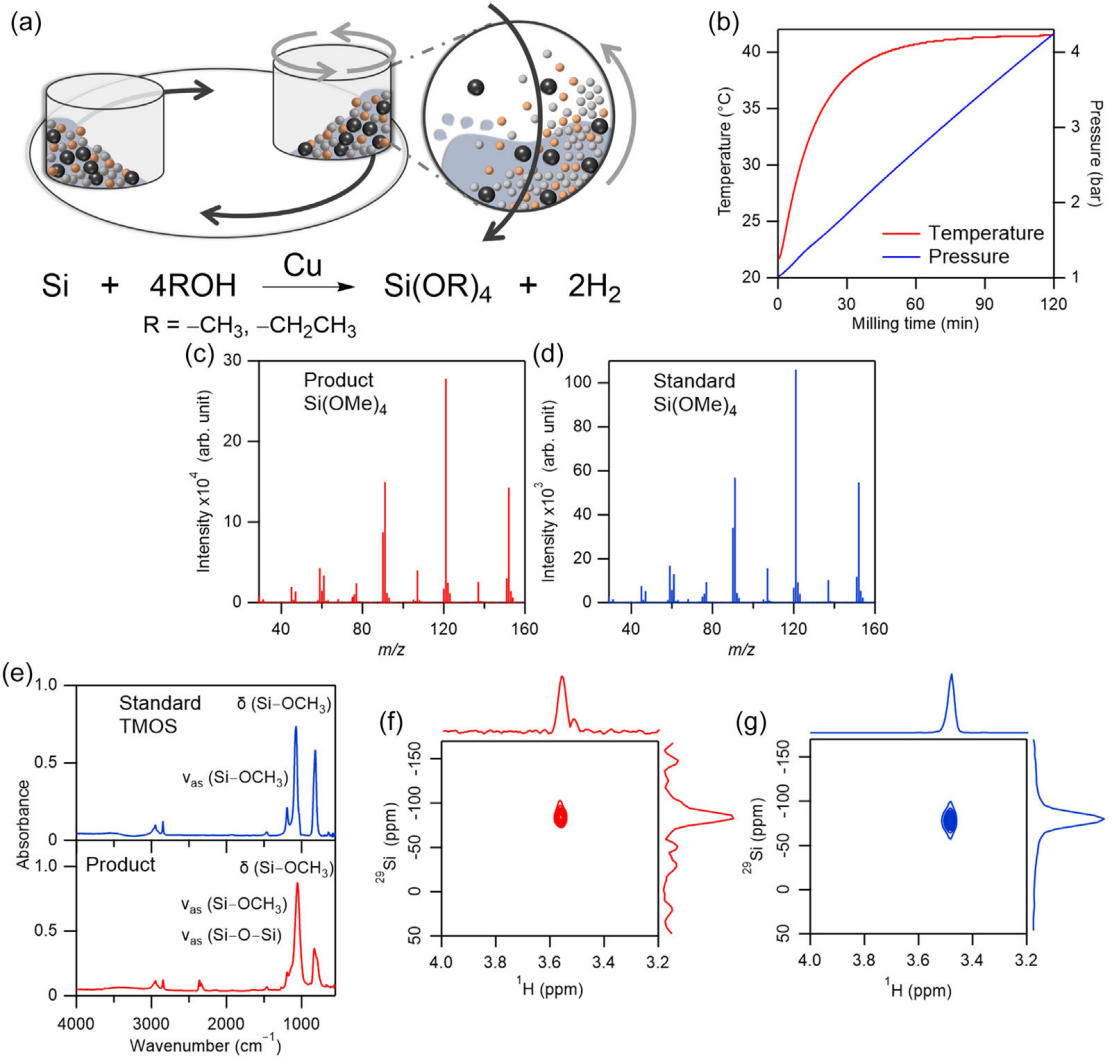
Si–alcohol mechanochemical reactions. (a) Schematic of mechanochemical synthesis of alkoxysilanes and hydrogen using a planetary ball mill. (b) Typical temperatures and pressures measured in situ, in the vessel, during the mechanochemical reactions. Mass spectra of (c) reaction products (methanol, Si–Cu powder mixture) and (d) a standard sample of TMOS as a reference. (e) FTIR spectra of a standard sample of TMOS and products. 2D NMR spectra of (f) reaction products and (g) a standard sample of TMOS. Reproduced with permission from ref. [22] (copyright 2022, American Chemical Society).

**FIGURE 7:**
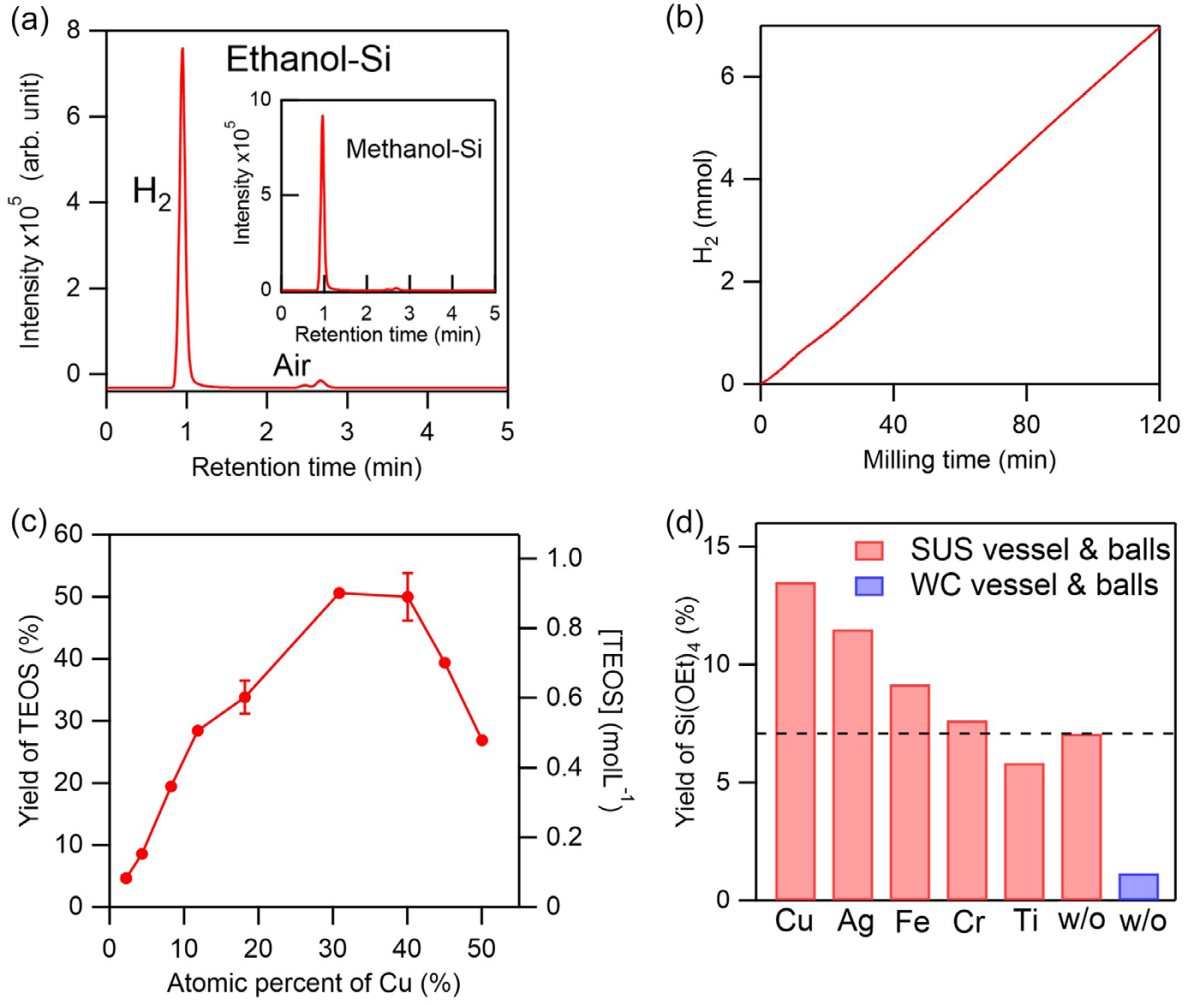
Detection and quantification of H_2_ product of Si–alcohol mechanochemical reactions. (a) Gas chromatograms of gas generated by the mechanochemical reactions between alcohols and Si powder. (b) Amount of H_2_ produced by the reaction between ethanol and Si vs. time. (c) Yield of TEOS vs. mole fraction of Cu in the Si–Cu mixture. (d) TEOS yields obtained using various metal catalysts. Red and blue bars represent the data for the stainless steel (SUS) and tungsten carbide (WC) milling media, respectively. Reproduced with permission from ref. [22] (copyright 2022, American Chemical Society).

We were able to demonstrate a direct, one‐step synthesis method for alkoxysilanes and hydrogen from Si powder and alcohols, using a planetary ball mill at 23°C–40°C without any heating, halogen, or HF (Figure 6a). This reaction proceeds solely owing to mechanical impact, and hence it represents the first room‐temperature, halogen‐free route for both alkoxysilane formation and hydrogen evolution (Figures 6 and 7a,b). Seven metal catalysts were examined under different milling conditions, and the products were analyzed using gas chromatography (GC), GC–mass spectrometry (GC–MS), nuclear magnetic resonance spectroscopy (NMR), Fourier‐transform infrared spectroscopy (FTIR), inductively coupled plasma optical emission spectroscopy (ICP‐OES), XRD, and energy‐dispersive X‐ray spectroscopy (EDS), i.e., Figure 6c–g. Remarkably, yields of up to 50% were achieved simply by milling Si powder in alcohol (Figure 7c) [22].

When methanol and ethanol were used for the mechanochemical reaction, tetramethoxysilane (TMOS) and tetraethoxysilane (TEOS) were produced, respectively, with H_2_ as a valuable coproduct (Figures 6 and 7a,b). Catalytic activity was found to be governed primarily by chemical rather than physical properties. Pretreatment of Si–Cu mixtures promoted the formation of silicide (Cu_3_Si), thereby enhancing yields, as Cu and Ag—both with a d^10^ electron configuration—exhibited the highest activity. Fe, Cr, Ni, and Mn, common components of stainless steel (SUS), showed moderate activity, indicating that even the milling vessel itself can function as a mechanococatalyst. In contrast, hard materials such as WC and Ti generated high local temperatures (∼290°C) but yielded little product due to their poor catalytic activity (Figure 7d). Contamination of the alkoxysilanes by the SUS medium was negligible (<50 ppm Fe/Cr), and the product purity was 99.9% [22].

This halogen‐ and HF‐free mechanocatalytic route thus enables the simultaneous production of high‐purity alkoxysilanes (99.9%) and hydrogen at close to room temperature, without any CO_2_ emissions or toxic byproducts. Higher yields were obtained by scaling up and increasing the Si powder amount due to the increased number of localized heating sites [22]. While extending the milling time further increases conversion, the pressure buildup owing to hydrogen evolution introduces practical challenges that must be addressed for continuous operation. Controlled scaling and further optimization of the selection of alcohols, catalysts, and reaction parameters could broaden the applicability of this method to industrial‐scale synthesis, in line with green‐chemistry and sustainable development goals.

## 6 Summary and Outlook

In summary, planetary ball milling has emerged as a powerful platform for sustainable chemical transformations, facilitating high‐efficiency hydrogen generation, visible‐light‐active TiO_2_ photocatalyst production, and halogen‐free alkoxysilane synthesis at room temperature. By harnessing localized extreme conditions—transient high pressures, temperatures, and microscale supercritical water domains—mechanochemical approaches overcome thermal limitations with minimal energy inputs and CO_2_ emissions. These processes coproduce value‐added materials, reuse industrial waste, and proceed in compact systems, all of which point to their potential for distributed energy generation and scalable manufacturing.

Building on these insights, mechanochemical hydrogen generation can be envisioned as a “hydrogen‐on‐demand” strategy, in which small, low‐power ball mills produce hydrogen at the point of use without requiring an external power source, high‐pressure storage, or centralized infrastructure. If realized, such portable systems could potentially be integrated into vehicles or compact devices, allowing them to generate hydrogen directly from water during operation, with the potential to redefine conventional paradigms of production, transport, and storage. Beyond mobility, these approaches may provide resilient, decentralized energy supplies for off‐grid or emergency applications, while simultaneously yielding valuable chemical byproducts for regional resource cycles.

However, several practical challenges must be addressed before the mechanochemical advances discussed in this Concept review can be implemented. These include optimizing milling parameters for long‐term stability, elucidating the roles of defects and enthalpy–entropy compensation in reaction pathways, establishing maintenance protocols for continuous operation, and scaling reactions while maintaining efficiency. Addressing these factors is essential to bridge the gap between laboratory demonstrations and robust real‐world deployment.

Overall, mechanochemistry—an emerging form of “planetary chemistry” driven by motion and impact—offers a generalizable, low‐carbon route bridging solid‐state fundamentals with practical technologies for sustainable fuels, chemicals, and functional materials. With continued progress on the remaining challenges, this technology may provide a realistic foundation for future distributed energy systems.

## Conflicts of Interest

The author declares no conflicts of interest.
